# The termite genus *Glyptotermes* (Isoptera, Kalotermitidae) from Paraguay

**DOI:** 10.3897/zookeys.1059.70500

**Published:** 2021-09-01

**Authors:** Rudolf H. Scheffrahn

**Affiliations:** 1 University of Florida, Fort Lauderdale Research & Education Center, 3205 College Avenue, Davie, Florida 33314 USA University of Florida Davie United States of America

**Keywords:** *
Glyptotermes canellae
*, *Glyptotermeshickmani* sp. nov., humid Chaco, imago, soldier, redescription

## Abstract

A 2012 termite expedition yielded the first species of *Glyptotermes* known from Paraguay, *G.hickmani***sp. nov.** and *G.canellae* (Müller, 1873), the latter previously known from Argentina and Brazil. Both are described based on the soldier and imago castes.

## Introduction

*Glyptotermes* Froggatt, with 127 species worldwide, is the most speciose genus in the termite family Kalotermitidae (Krishna et al. 2013). Now with 27 New World species (all Neotropical), *Glyptotermes* is the second most speciose kalotermitid genus after *Cryptotermes* Banks. Scheffrahn (2019a) reported the current New World range of *Glyptotermes* and provided the first records of this genus from Bolivia, Colombia, Ecuador, French Guiana, Guatemala, Honduras, Paraguay, Peru, and much of the Lesser Antilles. The paucity of *Glyptotermes* records from throughout much of the Caribbean Basin and South America has been shown to be caused by a field sampling bias toward non-kalotermitids (Scheffrahn et al. 2018).

*Glyptotermes* species have a rather high wood moisture requirement and, therefore, are not found in arid parts of the Neotropics and are likewise, not economically important. Although the imago morphology is quite conserved, the head capsules of *Glyptotermes* soldiers are variously adorned with protuberances and rugosities, and robust mandibles that facilitate their identification. I herein describe *Glyptotermeshickmani* sp. nov. from three localities in the humid Chaco of Paraguay and redescribe *G.canellae* (Müller, 1873) from the same region.

## Material and methods

**Figure 1.**
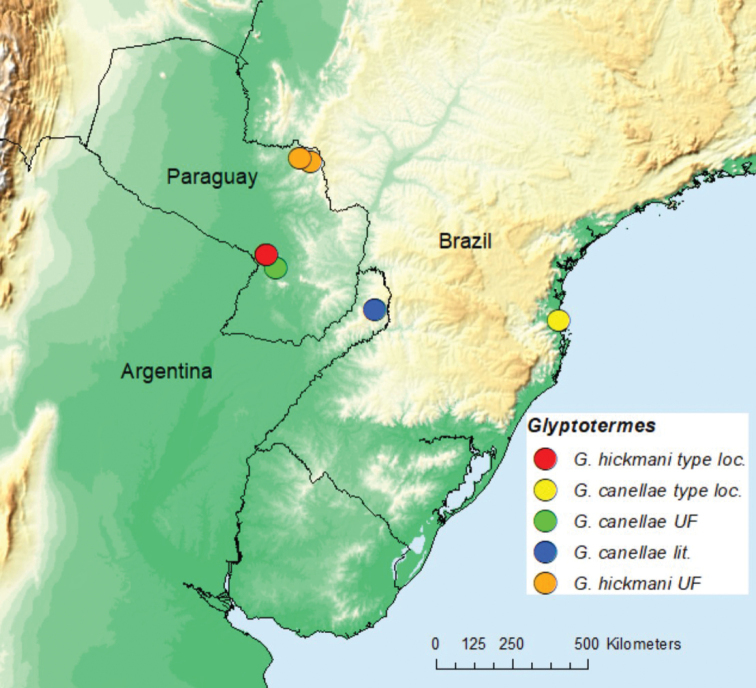
Distribution of *Glyptotermeshickmani* sp. nov. and *G.canellae* (Müller) in Paraguay and literature localities of *G.canellae* in Argentina and Brazil.

**Figure 2.**
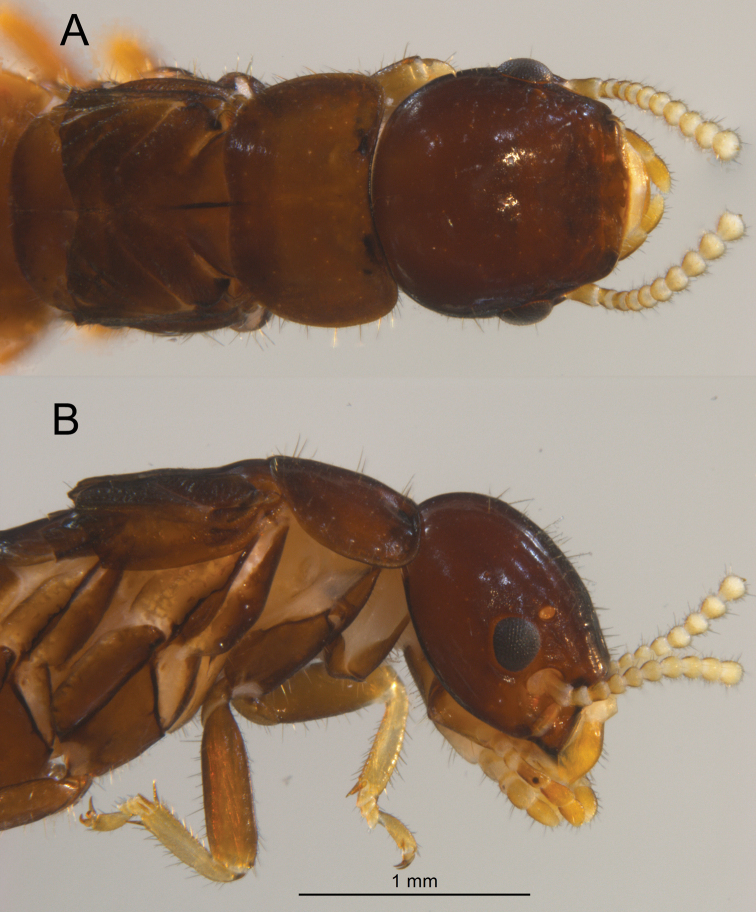
Dorsal (top) and lateral views of the *Glyptotermeshickmani* sp. nov. female dealate.

**Figure 3.**
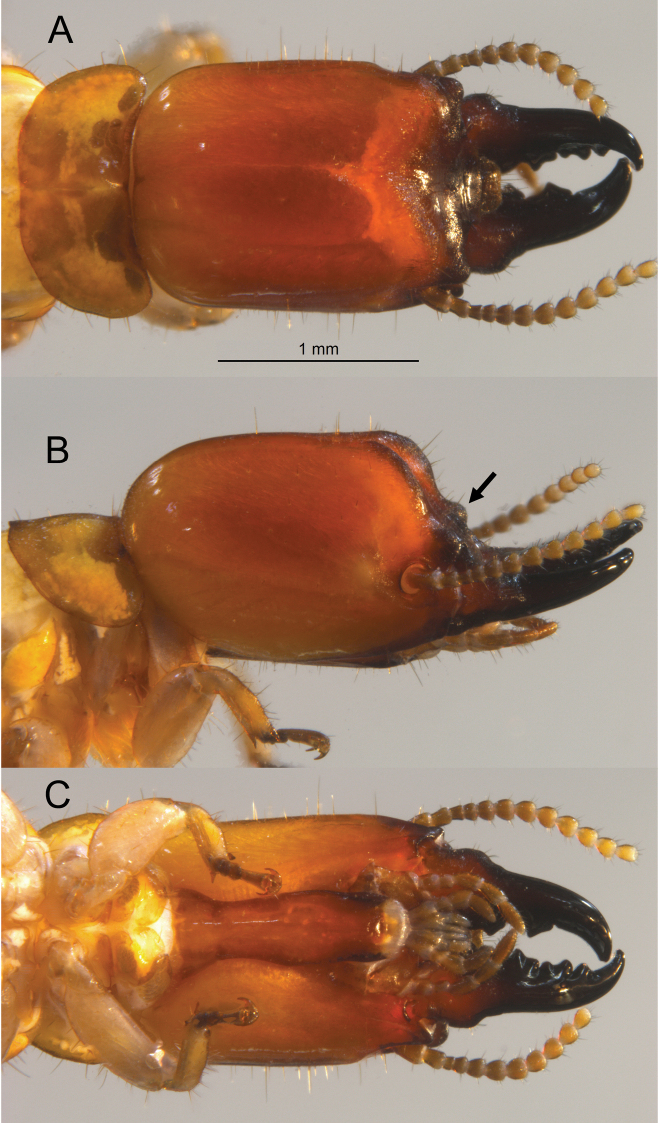
Dorsal (top) (**B**), lateral/oblique, and ventral views of the *Glyptotermeshickmani* sp. nov. soldier head capsule and pronotum. Arrow = frontal horn.

**Figure 4.**
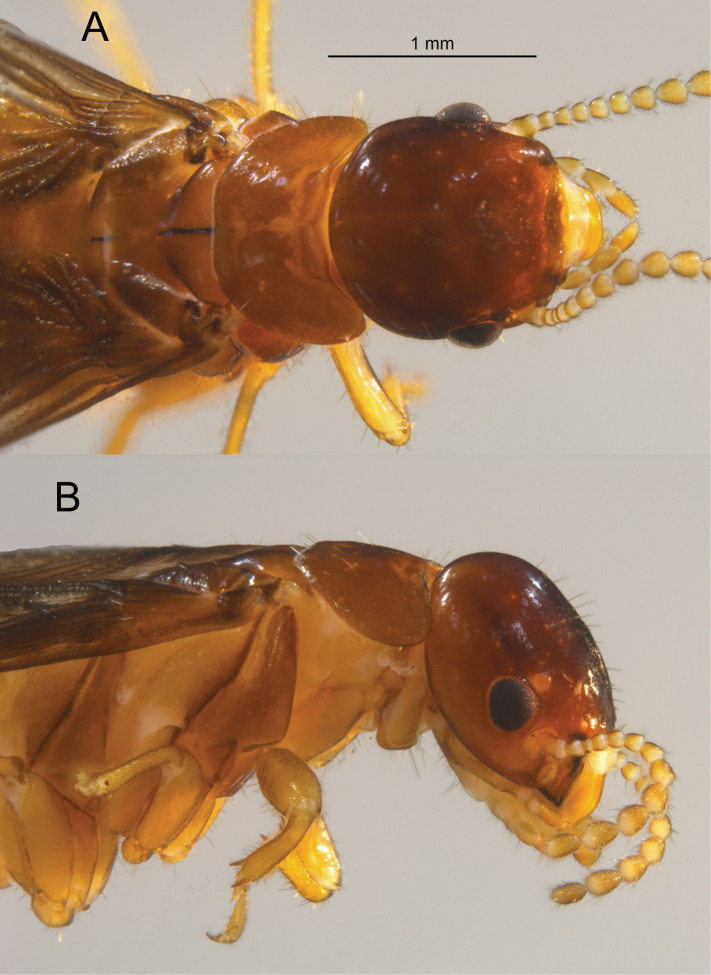
Dorsal (top) and lateral views of the *Glyptotermescanellae* (Müller) female alate.

**Figure 5.**
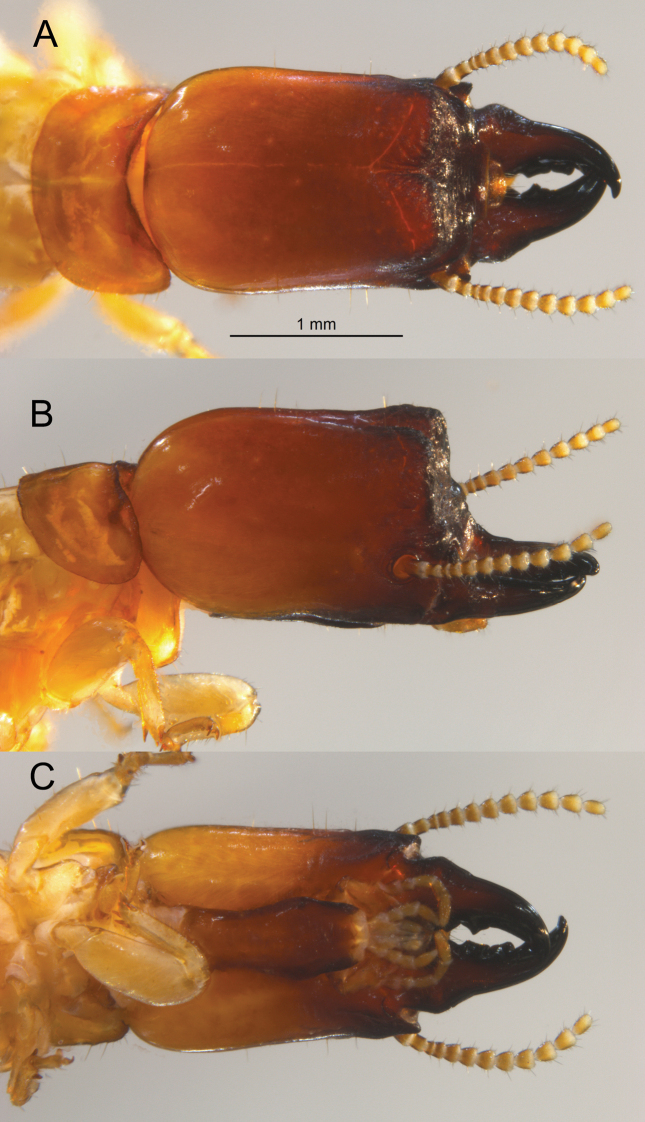
Dorsal (top), lateral/oblique, and ventral views of the *Glyptotermescanellae* (Müller) soldier head capsule and pronotum.

The distribution map (Fig. 1) was prepared using ArcMap 10.3 software (ESRI, Redlands, CA). Microphotographs (Figs 2–5) were taken as multi-layer montages using a Leica M205C stereomicroscope controlled by Leica Application Suite version 3 software. Preserved specimens were taken from 85% ethanol and suspended in a pool of Purell Hand Sanitizer to position the specimens on a transparent Petri dish background. Additional images of *Glyptotermes* are available at Scheffrahn (2019b).

## Taxonomy

### Glyptotermes hickmani sp. nov.

Taxon classificationAnimaliaBlattodeaKalotermitidae

A32A4091-456D-5D4A-B4B6-F4DAFE56D085

http://zoobank.org/99B790A9-DF50-49E9-9A75-F0D67FB8F3FA

**Figure 6.**
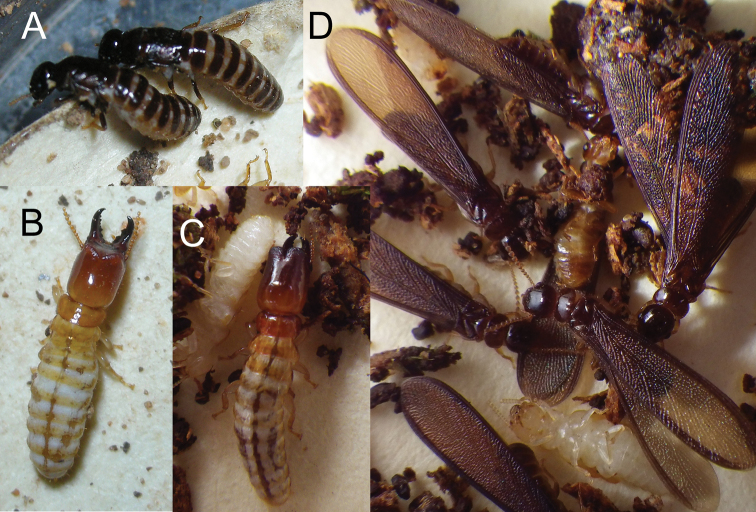
Live habitus of *Glyptotermeshickmani* sp. nov. imagos **A** and soldier **B** and *G.canellae* (Müller) soldier **C** and imagos **D**.

Figs 2 , 3 , 6 ; Tables 1 , 2

#### Diagnosis.

Among the ten South American *Glyptotermes* species for which the soldier is known, the *G.hickmani* sp. nov. soldier is closest in head width to *G.guianensis* (Emerson, 1925), but in lateral view, *G.guianensis* has angular frontal horns compared to the rounded frontal horns of *G.hickmani*. The frontal horns of the *G.hickmani* soldier are diagnostic.

#### Type locality.

Paraguay, Nueva Colombia.

#### Material examined.

***Holotype*** soldier: Paraguay, Nueva Colombia (-25.1747, -57.2876), elev. 115 m, 5JUN2012, R. Scheffrahn et al. (R. Scheffrahn, J. Chase, R. Hickman, J. Křeček, J. Mangold, A. Mullins), University of Florida Termite Collection (UFTC), Davie Florida, no. PA1265. ***Paratypes*.** Five additional soldiers, one queen, pseudergates/nymphs, same colony sample as holotype. One additional colony from type locality (same data), four soldiers, one king, one queen, and pseudergates/nymphs (PA1264). Paraguay, Cerra Cora (-22.6788, -55.9950), elev. 293 m, 29MAY2012, R. Scheffrahn et al., UFTC no. PA326 containing two soldiers and pseudergates/nymphs. Paraguay, 58 km W of Pedro Juan Caballero (-22.5600, -56.3006), elev. 328 m, 29MAY2012, R. Scheffrahn et al., UFTC no. PA446 containing one soldier and 12 pseudergates/nymphs.

#### Description.

***Dealated Imago*** (Fig. 2, Table 1). Head and pronotum castaneus brown (almost black in live habitus, Fig. 6A). Postclypeus hyaline; labrum light orange-brown. Fore wing scales concolorous with pronotum. Femora slightly lighter than pronotum; tibiae concolorous with labrum. In lateral view, vertex of head with 20–24 scattered setae of medium length. In dorsal view, pronotum with about 20 setae of variable length along each lateral margin; in lateral view, with setae in line with anterior and posterior margins; a few in middle. Eyes small, dark gray, occupying less than one-third the distance between vertex and genal margins; ellipsoid with rectate margin at antennal socket. Ocelli small, orange, elliptical; separated from eye by their narrow width. Antennae article formula 2 < 3 > 4 = 5. Pronotum wider than long, about as wide as head capsule without eyes; anterior and posterior margins with slight concavity, sides slightly convex. Arolia present.

**Table 1.** Measurements (mm) of *Glyptotermeshickmani* sp. nov. and *G.canellae* (Müller) imagos.

Characters	* G. hickmani *		* G. canellae *	
	(n = 4)		(n = 12)	
	mean	range	mean	range
Length of head to tip of labrum	1.38	1.35–1.44	1.36	1.30–1.40
Length of head to side base of mandibles	0.64	0.54–0.79	0.64	0.58–0.79
Width of head	1.15	1.11–1.19	1.18	1.14–1.19
Diameter of eye	0.26	0.25–0.30	0.27	0.25–0.28
Eye from lower margin	0.27	0.23–0.33	0.18	0.16–0.25
Length of ocellus	0.1	0.09–0.11	0.1	0.09–0.11
Median length of pronotum	0.62	0.58–0.65	0.57	0.53–0.60
Maximum length of pronotum	0.71	0.70–0.74	0.7	0.65–0.77
Width of pronotum	1.19	1.09–1.26	1.06	1.00–1.11
Length of hind tibia	0.91	0.86–0.96	0.87	0.79–0.91
Length of fore wing from suture			6.57	6.24–6.80
Width of fore wing			1.83	1.68–1.93

***Soldier*** (Fig. 3, Table 2). Head, in lateral view, grading from light orange at the cervical margin to dark brown at frontal horns (darker in live habitus, Fig. 6B). Mandibles concolorous with frons. Head capsule nearly cuboid; about as wide as high; with distinct narrowing near anterior fourth. Pronotum more than twice as wide as long, widest near front third; anterior margin weakly concave, posterior margin barely concave in middle. In dorsal and ventral views, setae on head and pronotum medium to long; about 4–5 short setae on basal hump of mandibles. In lateral view, setae less numerous except for setae on postmentum. Mandibles robust, form about two-thirds of total head length; curve apically near anterior half, humps at basal fourth evenly rounded beyond outer blades. Dentition as in Fig. 3. Frons bilobed; lobes rounded in lateral view; frontal furrow between lobes extends to postclypeus. Angle formed by vertex and frons about 75°; lateral view of frons punctuated by large, knob-like, frontal horns. Eye spots very faint. In ventral view, posterior margin of postmentum slightly wider than middle. Genal horns rounded from above, project slightly beyond narrowest point of head capsule. Antenna with 11–12 articles; formula 2 > 3 < 4 < 5. Femora swollen.

**Table 2.** Measurements (mm) of *Glyptotermeshickmani* sp. nov. and *G.canellae* (Müller) soldiers.

Characters	* G. hickmani *		* G. canellae *	
	(n = 9)		(n = 2)	
	mean	range	mean	range
Length of head to side base of mandibles	1.44	1.09–1.70	1.84	1.81–1.88
Width of head	1.22	1.00–1.32	1.37	1.32–1.42
Height of head	1.11	0.95–1.18	1.19	1.19–1.19
Length of left mandible	1.04	0.88–1.14	1.20	1.18–1.23
Maximum width of postmentum	0.38	0.35–0.40	0.51	0.49–0.53
Minimum width of postmentum	0.29	0.26–0.33	0.37	0.35–0.39
Length of postmentum	1.12	0.89–1.25	1.12	1.12–1.12
Median length of pronotum	0.54	0.40–0.61	0.59	0.58–0.60
Maximum length of pronotum	0.65	0.51–0.74	0.73	0.72–0.74
Width of pronotum	1.19	0.91–1.32	1.26	1.23–1.30
Length of hind tibia	0.72	0.61–0.79	0.88	0.88–0.88

#### Distribution.

See Fig. 1.

#### Etymology.

Named after Robert B. (Bob) Hickman, a friend and able termite collector, who joined us on the Paraguay expedition.

### Glyptotermes canellae

Taxon classificationAnimaliaBlattodeaKalotermitidae

(Müller, 1873)

01162141-9B85-5DAC-861A-229E8392DFD9

Figs 4 , 5 , 6 ; Tables 1 , 2

Müller, 1873, 334: Calotermes canellae ; imago described. Brazil.
Silvestri, 1901, 3: Calotermes lobicephalus ; soldier described with single measurement. Argentina.
Silvestri, 1903, 36–37: Calotermes lobicephalus ; soldier described with seven measurements and figured with simple line drawings. Argentina.
Holmgren, 1911, 56: Calotermes lobicephalus synonymized with C.canellae; imago, soldier figured with photographs.
See Krishna et al. (2013: 429) for complete synopsis and literature review.

#### Diagnosis.

Among mainland South American *Glyptotermes*, the soldier of *G.canellae* is the second largest after *G.hospitalis* (Emerson, 1925) from Guyana. The projecting genal horns of the *G.canellae* soldier are diagnostic.

#### Type locality.

Brazil: Santa Catarina: Itajaí.

#### Material examined.

Paraguay, S. Piribebuy (-25.5368, -57.0244), elev. 229, 27 May 2012, R. Scheffrahn et al., UFTC no. PA89 containing 2 soldiers, 12 alates, and many pseudergates. The types were not examined, but the soldier measurements in Silvestri (1903: 36) and photographs in Holmgren (1911; pl. 4, figs 14, 15) of *C.lobicephalus* are of excellent quality. The measurements agree with those in Table 2 and the photographs show the characteristically deeply bilobed frons and its steep angle from the vertex (Figs 5, 6C) that are unique for *Glyptotermes* soldiers of this region.

#### Redescription.

***Winged Imago*** (Fig. 4, Table 1). Head medium brown, pronotum light brown (castaneus in live habitus, Fig. 6D). Postclypeus hyaline; labrum light orange-brown. Fore wing scales concolorous with pronotum. Femora and tibiae concolorous with labrum. In lateral view, vertex of head with about 12 scattered setae of medium length. In dorsal view, pronotum with about 8–10 setae of various lengths along lateral margins; in lateral view, about 4–6 setae in line, each with anterior and posterior margins; a few in middle. Eyes medium small, dark gray, occupying about one-third the distance midway between vertex and genal margin; ellipsoid with rectate margin at antennal socket. Ocelli orange, nearly circular; one fifth their diameter from eye. Arolia present.

***Soldier*** (Fig. 5, Table 2). Head, in lateral view, grading from light orange at the cervical margin to dark brown at frons (darker in live habitus, Fig. 6C). Mandibles concolorous with frons. Head capsule much longer than wide; about as wide as high; in dorsal view, posterior wider than width at genal horns. Frontal horns absent. Pronotum more than twice as wide as long, widest near front third; anterior and posterior margins evenly curved, nearly parallel. In all views, setae sparse and uneven in length; no setae on basal hump of mandibles. Mandibles robust, form about one third of total head length; curve apically near anterior half, humps at basal fourth angular beyond outer blades. Dentition as in Fig. 5. Frons bilobed, lobes and frons rugose. Frontal furrow between lobes extends to middle of frons. Angle formed by vertex and frons about 90°; lateral view of frons with very small elevations near base; insufficient to constitute horns. Eye spots very faint. In ventral view, posterior margin of postmentum narrower than middle. Genal horns acute, visible from above, span narrowest part of head capsule. Antenna with 12 articles; formula 2 > 3 < 4 < 5. Femora swollen.

#### Distribution.

Argentine record of *G.canellae* from Torales et al. (1997) (Fig. 1).

## Discussion

Until now, no *Glyptotermes* species were known from Paraguay, while the following Paraguayan kalotermitids are reported: *Cryptotermeschacoensis* (Roisin, 2003), *Neotermesfulvescens* (Silvestri, 1901), *Rugitermesrugosus* (Hagen, 1858) in Scheffrahn (2019b), and *Tauritermestriceromegas* (Silvestri, 1901) in Scheffrahn and Vasconcellos (2020). The distributional range of *G.hickmani* is 340 km within the humid Chaco [tropical moonson biome per Koppen-Geiger; Kottek et al. (2006)] of Paraguay (Fig. 1). The range of *G.canellae* has been extended by 340 km within this same biome. Many species of South American *Glyptotermes* remain to be described (Scheffrahn 2019b). Our 2012 expedition to Paraguay also yielded a new *Neotermes* species, a new *Rugitermes* species, and many other new termite taxa.

## Supplementary Material

XML Treatment for Glyptotermes hickmani

XML Treatment for Glyptotermes canellae
